# Sociology, Social Class, Health Inequalities, and the Avoidance of “Classism”

**DOI:** 10.3389/fsoc.2019.00056

**Published:** 2019-07-05

**Authors:** Graham Scambler

**Affiliations:** ^1^Department of Sociology, University of Surrey, Guildford, United Kingdom; ^2^Institute of Population Health, University College London, London, United Kingdom

**Keywords:** social class, health inequalities, classism, class/command dynamic, institutional taming, foresight and action sociology

## Gender, Ethnicity and Class (and Its Proxies)

There is no doubting the causal impact of gender and race or ethnicity on health and health care. They are clearly implicated in the production and reproduction of health inequalities, and they no less clearly antedate the class-based stratification system of capitalism through all its phases. But, I contend, it is class that is of stand-out importance for a sociology of health inequalities. Sayer (2015, p. 170) explains why this is:

“*While gender and race inequalities are produced primarily by sexism and racism, class differences would persist even if the upper and middle classes were nice and respectful to the working class. The unequal distribution of property and the unequal division of labor would be largely unaffected. Class prejudice is common, but it's more a ‘response to' economic inequalities than a cause of them. By contrast, the enduring of sexism and racism would have a major impact on gender and race relations.”*

He continues:

“*Neoliberals – New Labor for example – can appear quite progressive about gender, race, sexuality, disability and condemn those who discriminate against people on these grounds. Unsurprisingly, the elephant in the room is economic inequalities or class difference. Though it never admits it, neoliberalism is a political-economic movement that seeks to legitimize widening economic inequalities and defend rentier interests above all others. Rentiers can live off others regardless of their gender, race, sexuality and so on.”*

Class has lost a degree of its salience for identity-formation in the individualized, “liquid” or relativized culture of contemporary financial capitalism, *but it has lost none of its structural force*. If it has a reduced impact “subjectively”, it has, I contend, enhanced its impact “objectively”.

With regard to health inequalities, the measures of class that have been deployed have been parsimonious proxies for the concepts familiar from classical sociology: Marx has all but disappeared and Weber has been tailored primarily to the study of trends in social mobility via Goldthorpe and his colleagues. I draw four conclusions from this. First, it seems apparent enough that the proxies or class routinely used to uncover, track and account for health inequalities, namely, the Registrar General's (RG) Classification of Occupations and the National Statistics Socio-Economic Classification (NS-SEC), substitute, respectively, occupational prestige and job characteristics for class as a social structure or relation (Bartley, 2017). The findings from this considerable array of investigations consistently reveal that the higher the likes of the prestige/rewards/security/authority/autonomy of people's jobs the better their chances of sustained good health and longevity. Marmot Review (2010) has even claimed, more controversially, that there is a discernible “social gradient” such that as employment grades increase, so too do the people's health prospects.

Second, it can nevertheless be inferred (in critical realist terminology, “retroduced”) from the findings of studies using these proxies that class as a pivotal and enduring structure or relation *must exist* for studies using the RG or NS-SEC classifications to deliver the findings they have long done and continue to do.

Third, to resort to class proxies like the RG and NS-SEC is to exclude the empirical consideration of class as a social structure or relation. More specifically—a hugely significant point in my view—it “absents” any possibility of investigating the putative causal salience of what has conventionally been seen as the “ruling class”, namely, that fraction of the top 1% that wields enormous and overriding social and political power (Scambler and Scambler, 2015). The 1% is swallowed alive and disappears from view and analysis in the RG and NS-SEC. As Coburn (2009, p. 44) has emphasized, the authors of extant investigations “seldom go far enough up the causal chain to confront the class forces and class struggles that are ultimately determinant.” I think this is an understatement and I shall focus later on the causally pivotal role of those *capital monopolists* comprising a tiny fraction of what Clement and Myles (1997) called the “capitalist executive” in the production and reproduction of health inequalities (see Scambler, 2018).

Fourth, I fully acknowledge that there are horses for courses. In other words, the NS-SEC, scrupulously derived from Weberian theory by Goldthorpe, is helpful, apt and functional for the study of changes in social mobility over time (see the excellent Budoki and Goldthorpe, 2019). But what it gives to sociology with one hand it takes away with the other.

## The Class/Command Dynamic

I have maintained elsewhere, first, that objective class relations are more not less potent in post-1970s financial capitalism than in the postwar welfare-state capitalism that preceded it; and second, that the capitalist executive in general, and the fragment of hard-core capitalist monopolists—those few major shareholders, rentiers, and CEOs—“players”—in our newly exacerbated global or transnational capitalist arena—in particular, have bought more policies from the state's power elite than was possible hitherto. The historian Landes (1998) has written that those with wealth have always bought those with power; and I maintain that they get more for their money in financial capitalism than they did in the postwar era. The rationale for capitalizing—an apt word—on this newfound purchasing power? The hard-core capital monopolists are, in Habermasian terms, totally, ineluctably “strategic”: they privilege accumulating capital to their personal advantage *above all else*. They are, as I have spelled out elsewhere, “focused autonomous reflexives” (Scambler, 2012). The bottom line, empirically, is that they have been able to purchase a greater range of policies favoring their own *personal and familial* accumulation of capital than hitherto, hence my reference to a novel class/command dynamic. This is critical not only for the deepening of wealth, income and—in their wake—health inequalities, but also for the ongoing dismantling of our NHS. Predictably, women, the dis-abled, the long-term sick and vulnerable, and those comprising class-disadvantaged segments of ethnic/racial minorities have suffered most.

This requires some elaboration. The main political plank of neoliberal ideology in the UK has been “austerity”. This discourse has provided cover for a sustained attack on working-class interests and well-being that has significantly enriched the capitalist executive in general and the—now super-rich—capital monopolists in particular. Other principal beneficiaries include those working for and with the capitalist executive and the power elite of the state. I have elsewhere defined the UK's “governing oligarchy” (though some, like Sayer, prefer the term “plutocracy”) as consisting of the hard-core of the transnational capitalist executive plus the nation-state's power elite (Scambler, 2018).

Working-class health and longevity has been measurably damaged by the strangulation of those capital or “asset flows” known to be conducive to good health. These can be summarized as follows:

- **Biological (or body) assets** can be affected by class relations even prior to birth. Low-income families, for example, are more likely to produce babies of low birthweight; and low birthweight babies carry an increased risk of chronic disease in childhood, possibly in part through biological programming;
- **Psychological assets** yield a generalized capacity to cope, extending to what is increasingly conceptualized as “resilience.” In many ways the “vulnerability factors” that Brown and Harris (1978) found reduced working-class women's capacity to cope with life-events salient for clinical depression are class-induced interruptions to the flow of psychological assets;
- **Social assets** have come to assume pride of place in many accounts of health inequalities and feature strongly in the work of Marmot and Wilkinson. The terms social assets or “social capital” refer to aspects of social integration, networks, and support. The political use to which social capital is sometimes put should not lead to its neglect;
- **Cultural assets** or “cultural capital” are initially generated through processes of primary socialization and go on to encompass formal educational opportunities and attainment. Class-related early arrests to the flow of cultural assets can have long-term ramifications for employment, income levels, and therefore health;
- **Spatial assets** have been shown to be significant for health by area-based studies. These have documented that areas of high mortality tend to be areas‘ with high rates of net out-migration; and it tends to be the better qualified and more affluent who exercise the option to move;
- **Symbolic assets**, representing the variable distribution of social status or “honor”, are known to impact on health via people's (sense of) social position, especially relative to those others who comprise their reference groups;
- **Material assets** refer to “relative deprivation” due to impoverishment and meager standard of living. The relevance of material assets for health and longevity has long been stressed, although the mechanisms linking low income with health remain much debated.

The process is as follows: concomitant with the enhanced class-driven character of government policy are a series of shifts of direction, such as part-time, transitory and insecure employment, extending to zero hours contracts; deteriorating workers' rights and conditions; the sidelining of trade unions; the ending of final salary pension schemes and the increase in the age of retirement; benefit cuts, culminating in the rolling out of Universal Credit; the defunding of local government services and, to an extreme degree, social care; NHS cuts and privatization; and so on.

The net effect of this targeted action against the welfare state and the working class, carried out under the aegis of austerity and a political rather than economic choice, is to severely reduce the flows of health-related assets for many people. Although there can be compensation between asset flows—a strong flow of social assets can for example mitigate the negative effects of a reduction in the flow of material assets following a job loss—the research suggests that reductions tend to cluster across flows, and also that reduced flows at critical junctures of the lifecourse, like childhood, can have severe deleterious and long-term effects. So there is an empirically traceable causal chain stretching from the boardroom decisions of big bosses and shareholders through elite career-oriented politicians to the bodies and minds of the most disadvantaged and vulnerable citizenry.

## Is Sociology Being “Tamed?”

I have built on Burawoy's (2005) explication of “four sociologies” by appending a further two. The resulting six sociologies are outlined here, together with ideal types of theory and of practitioner:

1. Professional sociology—scholar—cumulative theory
2. Policy sociology—reformer—utilitarian theory
3. Critical sociology—radical—meta-theory
4. Public sociology—democrat—communicative theory
5. Foresight sociology b- visionary—speculative theory
6. Action sociology—activist—strategic theory

It is in my view the responsibility of the community of sociologists as a whole to ensure that all six bases are covered, not that every sociologist should attempt all six. I will focus here on the import of foresight and action sociology for any consideration of health inequalities. Foresight sociology develops speculative theory (i.e., anticipates “alternative futures,” whether societal or institution-based) and is practiced by visionaries. Action sociology deploys strategic theory (i.e., is oriented to accomplishing change) and is conducted by activists. In the most general terms, sociologists as visionaries might focus on concrete ways in which what are commonly called “social determinants” of health and disease and of their unequal distributions might be so amended, rejigged, or subverted as to ensure greater equality of opportunity and outcome. Sociologists as activists would represent theories and research emanating from the scholars of professional sociology and the reformists of policy sociology in civil society and the public sphere of the lifeworld, *and go on to engage with and contest the dirty politics of their neglect and ideological dismissal* often conducted through the medium of right-wing think tanks unwilling to identity their sponsors.

To fail to convert professional, policy and critical sociology—via public sociology—into foresight *and* action sociology is to my mind to acquiesce, qua community, in the taming of the discipline. In my *Sociology, Health, and the Fractured Society* I illustrate further ways in which sociology can, and in my view is, being tamed (Scambler, 2018). I also offer sets of interrogations of population health and health inequalities pertinent to each of my six sociologies. Toward the end of this short piece I proffer another quartet of themes I think it interesting and important to pursue.

- With regard to sociology's putative taming, how does the availability of funding for health inequalities research break down by issue and focus?
- Precisely how, and how convincingly, does research into “class and health” via proxy measurements of class like the RG and NS-SEC warrant retroductive (or more rarely in qualitative research abductive) inference to the “reality” of class as a structure or relation impacting as a pivotal causal mechanism for sociological explanations of health inequalities?
- Does the extant body of (largely positivistic) research purporting to address the linkage between class and health—but in fact “absenting” class as a structure/relation—in effect function as a “protective belt” around financial capitalism's justificatory neoliberal ideology by absorbing or deflecting attention from what *should be* sociology's true and enduring point of departure toward subsidiary concerns?
- How, figuration by figuration, or context by context, does class as defined in this short paper fare as a causal mechanism salient for health inequalities *in comparison with those of gender and ethnicity/race*? While I make a case here for class as prepotent in the figuration/context of a British nation state precariously lodged in a capitalist world system, its causal power, as intersectionalists rightly insist, is obviously far from ubiquitous.

I was a fortunate babyboomer with a degree of latitude to negotiate “free time” to ask personal and inconvenient—that is, genuinely sociological—questions. Equivalent negotiations have indubitably grown tougher. *But*: (a) it actually wasn't quite as easy for us babyboomers as is often somewhat ritualistically assumed, and (b) it remains crucial that *we*, the sociological community, as a whole, *and as individual members of that community*, hold our nerve and ground (Scambler, 1996).

## Author Contributions

The author confirms being the sole contributor of this work and has approved it for publication.

### Conflict of Interest Statement

The author declares that the research was conducted in the absence of any commercial or financial relationships that could be construed as a potential conflict of interest.

## References

[B1] Bartley M. (2017). Health Inequality: An Introduction to Theories, Concepts and Methods, 2nd Edn. Cambridge: Polity Press.

[B2] Brown G. Harris T. (1978). Social Origins of Depression. London: Tavistock. 10.1017/s0033291700018791 724871

[B3] Budoki E. Goldthorpe J. (2019). Social Mobility and Education in Britain. Cambridge: Cambridge University Press.

[B4] Burawoy M. (2005). For public sociology. Am. Sociol. Rev. 70, 4–28. 10.1177/000312240507000102 15926908

[B5] Clement W. Myles J. (1997). Relations of Ruling: Class and Gender in Post-Industrial Societies. Toronto: McGill Queen's University Press.

[B6] Coburn D. (2009). “Inequality and health,” in Morbid Symptoms: Health Under Capitalism, eds Panitch L. Leys C. (Pontypool: Merlin Press), 39–58.

[B7] Landes D. (1998). Wealth and Poverty of Nations. London: Little, Brown & Co.

[B8] Marmot Review (2010). Fair Society, Healthy Lives: Strategic Review of Health Inequalities in England Post-2010. London: Department of Health.

[B9] Sayer A. (2015). Why We Can't Afford the Rich. Bristol: Policy Press.

[B10] Scambler G. (1996). The ‘project of modernity' and the parameters for a critical sociology: an argument with illustrations from medical sociology. Sociology 30, 567–581.

[B11] Scambler G. (2012). “Archer, morphogenesis and the role of agency in the sociology of health inequalities,” in Contemporary Theorists for Medical Sociology, ed Scambler G. (London: Routledge), 131–149.

[B12] Scambler G. (2018). Sociology, Health and the Fractured Society: a Critical Realist Account. London: Routledge.

[B13] Scambler G. Scambler S. (2015). Theorizing health inequalities: the untapped potential of dialectical critical realism. Soc. Theory Health 13, 340–354. 10.1057/sth.2015.14

